# Root exudates and rhizosphere soil bacterial relationships of *Nitraria tangutorum* are linked to k-strategists bacterial community under salt stress

**DOI:** 10.3389/fpls.2022.997292

**Published:** 2022-08-31

**Authors:** Yaqing Pan, Peng Kang, Min Tan, Jinpeng Hu, Yaqi Zhang, Jinlin Zhang, Naiping Song, Xinrong Li

**Affiliations:** ^1^Shapotou Desert Research and Experiment Station, Northwest Institute of Eco-Environment and Resources, Chinese Academy of Sciences, Lanzhou, China; ^2^College of Biological Sciences and Engineering, North Minzu University, Yinchuan, China; ^3^College of Pastoral Agriculture Science and Technology, Lanzhou University, Yinchuan, China; ^4^Breeding Base for Key Laboratory Land Degradation and Ecological Restoration in Northwest China, Ningxia University, Yinchuan, China

**Keywords:** *Nitraria tangutorum*, drought or salt stress, root exudates, rhizosphere bacteria, co-occurrence network, k-strategists

## Abstract

When plants are subjected to various biotic and abiotic stresses, the root system responds actively by secreting different types and amounts of bioactive compounds, while affects the structure of rhizosphere soil bacterial community. Therefore, understanding plant–soil-microbial interactions, especially the strength of microbial interactions, mediated by root exudates is essential. A short-term experiment was conducted under drought and salt stress to investigate the interaction between root exudates and *Nitraria tangutorum* rhizosphere bacterial communities. We found that drought and salt stress increased rhizosphere soil pH (9.32 and 20.6%) and electrical conductivity (1.38 and 11 times), respectively, while decreased organic matter (27.48 and 31.38%), total carbon (34.55 and 29.95%), and total phosphorus (20 and 28.57%) content of *N. tangutorum* rhizosphere soil. Organic acids, growth hormones, and sugars were the main differential metabolites of *N. tangutorum* under drought and salt stress. Salt stress further changed the *N. tangutorum* rhizosphere soil bacterial community structure, markedly decreasing the relative abundance of Bacteroidota as r-strategist while increasing that of Alphaproteobacteria as k-strategists. The co-occurrence network analysis showed that drought and salt stress reduced the connectivity and complexity of the rhizosphere bacterial network. Soil physicochemical properties and root exudates in combination with salt stress affect bacterial strategies and interactions. Our study revealed the mechanism of plant–soil-microbial interactions under the influence of root exudates and provided new insights into the responses of bacterial communities to stressful environments.

## Introduction

The plant root system is the interface for material exchange between the plant and soil ecosystem. As the life carrier between plants and the soil environment, root exudates play a prominent role in nutrient absorption and cycling (Mommer et al., 2016; Sokol et al., 2019). Root exudates, mainly including sugars, amino acids, organic acids, phenolic acids, fatty acids, sterols, proteins, and growth factors, can be divided into high and low molecular weight (Baetz and Martinoia, 2014; Wu et al., 2014; Canarini et al., 2019). Plants are often subjected to various biotic and abiotic stresses throughout their life cycle and respond by secreting different bioactive compounds through their roots (Badri and Vivanco, 2009). The composition and quantity of plant root exudates increase considerably under abiotic stress, and their organic acid content markedly increases under water stress (Vives-Peris et al., 2018). Furthermore, salt and other abiotic stressors can induce the synthesis and accumulation of plant secondary metabolites such as polyphenols (Zhou et al., 2018). Previous studies have shown that root exudates serve as the medium for co-evolution between plants and microorganisms and can promote the interaction between them by regulating the plant rhizosphere micro-environment to cope with various biotic and abiotic stresses (Pétriacq et al., 2017; Gargallo-Garriga et al., 2018; Guyonnet et al., 2018; Karlowsky et al., 2018; de Vries et al., 2019).

Soil microbial communities are closely associated with plant roots and participate in biogeochemical cycles, thus shaping the structure and function of terrestrial ecosystems (Bardgett and van der Putten, 2014; Fierer, 2017). The interaction between plant root exudates and rhizosphere microorganisms is an important process. For example, plant roots secrete various secondary metabolites affecting rhizosphere microorganism species, quantity, and distribution (Jenkins et al., 2017). Amino acids, vitamins, and carbohydrates in plant root exudates provide a basis for microbial growth and substrate decomposition (Zhalnina et al., 2018). Furthermore, some allelochemicals in root exudates can inhibit the assembly process of rhizosphere microbial communities (Xia et al., 2016). Soil moisture, vegetation type, and climate shape plant rhizosphere microbial communities through changes in plant rhizosphere metabolites (Kong et al., 2018). Plant root exudates and environmental selection drive the change in rhizosphere microbial community structure (Schleuning et al., 2015). Given the ubiquity, diversity, and plasticity of microorganisms, it is important to explore plant–soil-microorganism interactions mediated by root exudates and to have an in-depth understanding of microbial interactions and functions.

In the past decade, the diversity and complexity of microbial communities have made it urgent to understand the construction and characteristics of microbial communities (Duran et al., 2018; Rottjers and Faust, 2018). With the rapid development of high-throughput sequencing technology, microbial community data have become more accurate. The network of microbial interactions provides a multidimensional and more complete view of ecosystems. These networks are closely related to ecosystem functions and play a key role in maintaining biodiversity (Wardle, 2006). Therefore, the application of microbial network analysis to identify alternative community states and ecological niches has become a common tool for studying the structure of microbial communities (Xiao et al., 2017; Kumar et al., 2019). In microbial co-association networks, nodes represent species and edges represent potential interactions between species (Bahram et al., 2014; Ma et al., 2016). There is growing evidence that the properties of ecological networks, such as soil pH (Banerjee et al., 2019), soil water availability (Ma et al., 2020; Hernandez et al., 2021), and soil nutrients (Shi et al., 2020), may represent interactions between coexisting organisms. A microbial community is a well-constructed complex ecological network in which microorganisms collaborate and interact to maximize their ecological functions (Toju et al., 2018; Banerjee et al., 2019).

Given the large number of soil bacteria, the concept of r-and k-strategists can be applied to the study of soil bacterial ecology to understand bacterial interactions (Fierer et al., 2007; Pascault et al., 2013). R-strategists preferentially consume unstable soil nutrients and maximize their intrinsic growth rate when resources are abundant (Kielak et al., 2009). Contrastingly, k-strategists show a slower growth rate and compete for survival under nutrient deficiency (Meyer, 1994). Studies have shown that in the ecological classification of soil bacteria, Betaproteobacteria, Gammaproteobacteria, Deltaproteobacteria, and Bacteroidota are r-strategists, and Alphaproteobacteria and Acidobacteriota are k-strategists (Klappenbach et al., 2000; Lee et al., 2008; Senechkin et al., 2010; Gu et al., 2018). Microbial communities often respond to environmental stress considering their growth, reproduction, competition, and adaptation strategies. However, there is still a limited understanding of the effects of root exudates on the adaptation strategies of plant rhizosphere bacterial communities under environmental stress.

Soil microorganisms are essential for maintaining the structure and function of terrestrial ecosystems. Many environmental factors (soil pH, moisture, and nutrient availability) and plant root exudates directly or indirectly influence soil microbial communities (Bardgett and van der Putten, 2014; Delgado-Baquerizo et al., 2016; Sayer et al., 2017; Su et al., 2020). However, the mechanisms by which plant root exudates influence rhizosphere microbial communities of the dominant plant species in the desert steppe ecosystems in China are not fully understood. *Nitraria tangutorum* is a typical desert shrub with strong resistance to salinity and drought, and widely distributed in arid areas of Northern China (Luo et al., 2008; Ni et al., 2015; Yan et al., 2018). It is of great significance for sand fixation, soil improvement and has very important ecological functions (Liu et al., 2016). Its fruit is also an important nutritional health food resource with high potential economic value (Kang et al., 2015). In this study, we analyzed the differences in root exudates and microbial communities of *N. tangutorum* under short-term drought and salt stress through controlled experiments. We proposed that there are differences in the root exudates of *N. tangutorum* under short-term drought and salt stress, and that the differences in root exudates affect bacterial survival strategies and interactions.

## Materials and methods

### Plant materials and treatments

In this study, *N. tangutorum* was obtained from the Lianhuachi Lake in Dingbian County (37°36′20′N, 107°20′25′ E), Shaanxi Province, China, in May 2021. Lianhuachi Lake is 1,310 m above sea level and covers 0.61 km^2^. The average annual precipitation is less than 200 mm. *Nitraria tangutorum* with the same growth were transplanted into pots (Φ = 33 cm, height = 20 cm) with sand and vermiculite at a ratio of 4: 1 and placed in a greenhouse. The growth conditions in greenhouse were controlled to maintain a temperature of 26/22°C (day/night), a photoperiod of 16/8 h (light/dark). The plants were watered with 1/2 Hoagland solution and subjected to drought and salt stress after 2 months. In “CK” group, the SWC was maintained at 50% of FWC by irrigated with 1/2 Hoagland solution. In “Drought” group, the SWC was maintained at 30% of FWC by irrigated with 1/2 Hoagland solution. All these values were subsequently maintained constantly by irrigated with corresponding solutions every 5 days for 30 days. In “Salt” group, the SWC was maintained at 50% of FWC by 1/2 Hoagland solution containing 200 mM NaCl, and the treatment solutions were renewed every 5 days to keep constant NaCl concentration (Kang et al., 2016; Pan et al., 2016; Guo et al., 2020).

### Sample collections

After 30 days of treatment, *N. tangutorum* were removed from the pots, and the rhizosphere soil was collected; 27 soil samples were collected, including nine CK, nine drought, and nine salt samples. For each treatment, the soil probe was washed with 75% ethanol before collection and gloves were changed before collection. The soil cores were then placed in a sterile centrifuge tube and taken to the laboratory in an ice box (Pan et al., 2021). After sieving and removing surface vegetation and litter, the soil samples were divided into three parts: one for high-throughput sequencing, one for analysis of physicochemical properties, and the other was frozen for metabolite extraction. Soil samples were air-dried for the determination of soil physicochemical properties, and fresh soil samples were used for the determination of soil microorganisms and root exudates. We took 100 g of fresh soil for extraction and collection of root exudate. Firstly, 500 ml deionized water was added, and then centrifuged for 5 min (20°C，8,000 r/min) after shock extraction for 3 h, and then the supernatant was extracted for filtration. After that, the water was spin-dried at 35°C with the vacuum rotary evaporator, poured on the tin foil until the methanol completely volatilized, and washed repeatedly 2–3 times; and finally stored at −80°C to be tested.

### Characterization of physicochemical properties of rhizosphere soil samples

Soil water content (SWC) was measured using the weighing method (Li et al., 2020), soil pH was determined using a pH meter (Mettler S220; Mettler Toledo Solutions, Greifensee, Switzerland), and soil electrical conductivity (EC) was measured using a specific conductivity meter (Leici DDS-307A, Shanghai Leici Instruments, Shanghai, China) with the soil-water ratio of 1:5 (Wang et al., 2019). After the collected soil samples were air-dried, the soil total carbon (TC), total nitrogen (TN), and total phosphorus (TP) were determined using atomic absorption spectrometry with an atomic absorption spectrophotometer (iCE 3,500, Thermo Fisher Scientific, Waltham, MA, United States; Bettinelli et al., 2000; Postma et al., 2016). Soil total organic carbon (TOC) was determined by dichromate oxidation and ammonium ferrous sulfate titration (Gul et al., 2015).

### Determination of root metabolites

Nucleic acids were extracted from each sample at the end of the treatment. Plant roots were carefully rinsed with sterile deionized water and soaked in 1,000 ml of sterile deionized water for 8 h, according to Luo et al. (2014). The extract was stored in liquid nitrogen and sent to Novogene Bioinformatics Technology Co. Ltd., Tianjin, China, for the determination of plant root exudates using liquid chromatography-mass spectrometry (LC–MS; Ultimate 3000 LC, Q Exactive; Thermo Fisher Scientific) on a Hyper Gold C18 column [100 mm × 2.1 mm, at 1.9 μm, (Thermo Fisher Scientific)]. Metabolites were detected in positive and negative ion modes. Before sample detection, equal amounts of samples were extracted from 27 root exudates and mixed into a quality control (QC) sample. The total ion flow chromatograms of the QC samples were overlapped to verify the reproducibility of the retention time of the same substance. Compound Discoverer Software (Thermo Fisher Scientific) was used to extract and preprocess the LC–MS detection data, including retention time, molecular weight, sample name, and peak intensity. Subsequently, the ionic characteristics were combined with three predicted components databases (Predicted Compositions, mzCloud Search, and ChemSpider Search) to determine the compound information. Finally, the peak value was converted to the peak value per unit mass using the dry weight of the secretions.

### Soil DNA extraction and PCR amplification

The rhizosphere soil bacterial community was examined using Illumina MiSeq sequencing kits (Illumina, San Diego, CA, United States). Total genomic DNA of *N. tangutorum* rhizosphere soil was extracted using the cetyltrimethylammonium bromide/sodium dodecyl sulfate method. The DNA concentration and purity were examined on a 1% agarose gel. Considering the concentration, DNA was diluted to 1 ng/μl using sterile water. All PCR reactions were carried out using 15 μl of Phusion High-Fidelity PCR Master Mix (New England Biolabs, Ipswich, MA, United States), 0.2 μM of forward and reverse primers, and approximately 10 ng template DNA. Illumina MiSeq sequencing libraries (Illumina) for bacteria were prepared *via* PCR amplification of the V3–V4 hypervariable regions of the bacterial 16S rRNA gene using the primers 338F (5′-ACTCCTACGGGAGGCAGCAG-3′) and 806R (5′-GGACTACHVGGGTWTCTAAT-3′) using a GeneAmp 9700 PCR thermocycler (Applied Biosystems, Waltham, MA, United States; Caporaso et al., 2012; Langille et al., 2013). The thermal cycling conditions were as follows: an initial denaturation at 98°C for 1 min, followed by 30 cycles of denaturation at 98°C for 10 s, annealing at 50°C for 30 s, and elongation at 72°C for 30 s, and finally extension at 72°C for 5 min (Haas et al., 2011). The purified amplicons were pooled in equimolar concentrations and paired-end sequenced on an Illumina MiSeq platform (Illumina) according to the standard protocols of Novogene Bioinformatics Technology Co., Ltd. The 16S rRNA gene sequences obtained in this study have been submitted to the NCBI Sequence Read Archive (SRA) database with the serial number PRJNA855333.

### Sequence processing and statistical analysis

PE libraries were constructed using a NEXTflex Rapid DNA-SEQ Kit (Bioscience, South San Francisco, CA, United States), and an Illumina MiSeq PE300 platform (Illumina) was used for sequencing. Trimmomatic software (Illumina) was used for quality control of Illumina MiSeq sequencing original sequences. FLASH 1.2.11;^1^ software (Illumina) was used for stitching (Magoč and Salzberg, 2011). UPARSE 7.1 software was used for amplicon sequence variants (ASVs) clustering analysis of the sequences (similarity 97%), and UCHIME software was used to remove chimeras (Caporaso et al., 2012). Each sequence was annotated for species classification by the ribosomal database project classifier and compared with the Silva database (SSU128) at a confidence threshold of 0.7 (Quast et al., 2013). All calculations were performed by sub-sampling each sample to an equivalent sequence of 49,556 (Ye et al., 2017).

The QIIME program (2.0) was used to calculate the alpha diversity indices (Shannon and Chao1; Kuczynski et al., 2012). The linkET^2^ package in R software (Version 4.1.0) was used to calculate and visualize the correlation between soil physicochemical properties and soil bacterial α-diversity. Canonical correlation analysis (CCA) was applied to distinguish the soil physicochemical properties, differential metabolites community, and bacterial phyla under drought and salt stress (Ramette, 2010). A non-metric multidimensional scale (NMDS) based on the Bray–Curtis distance matrix and ANOSIM test with 9,999 permutations were used to illustrate beta diversity. Differences in community composition between sample groups were analyzed using the vegan package (Pan et al., 2021). The ggalluvial package in R software was used to describe changes in bacteria (top 10 phyla and top 20 genera with the highest abundance). A co-occurrence network was constructed for each treated sample, and coefficients of all possible Spearman correlations among ASVs in all samples were calculated using the psych package (Berry and Widder, 2014; R Core Team, 2020). Cytoscape (3.7.1) was used for network visualization, and the number of ASVs at the phylum level was statistically analyzed. The bacterial phyla or classes for r-and k-strategists were visualized using the ggplot2 package. The psych package was used to calculate the correlation between differential metabolites and soil properties, which was then visualized using the ggplot2 package. We used the fold change value and value of *p* to screen for significantly upregulated or downregulated differential metabolites and visualized them using the ggplot2 package.

SPSS version 25.0 (SPSS Inc., Chicago, IL, United States) was used for one-way ANOVA of soil physicochemical data, and Duncan’s multiple range test was used to identify the significant differences between means at a 5% significance level. All data are presented as the mean ± SE (*n* = 9; Pan et al., 2021).

## Results

### Relationship between rhizosphere soil bacterial community diversity and physicochemical properties under drought and salt stress

The physicochemical properties of *N. tangutorum* rhizosphere soil were different under drought and salt stress. Salt and drought stress markedly increased rhizosphere soil pH and EC of *N. tangutorum*. Under salt stress, pH and EC were 20.55% and 5.2-fold higher than those of CK, respectively. Conversely, salt and drought stress decreased rhizosphere SOM, TC, and TP contents. Rhizosphere SOM, TC, and TP contents decreased by 38.03, 52.85, and 24.39%, respectively, under drought stress, and by 45.62, 42.73, and 39.48%, respectively, under salt stress, compared with that in CK (*p* < 0.05; Supplementary Table 1).

**Figure 1:**
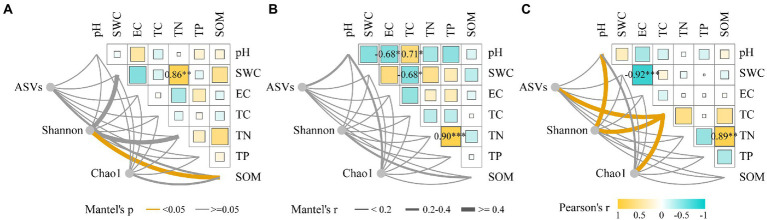
Correlation between environmental factors and alpha diversity indices of *Nitraria tangutorum* rhizosphere soil under different stress (**A**: CK; **B**: Drought; and **C**: Salt). Pairwise comparisons of environmental factors are shown with a color gradient denoting Pearson’s correlation coefficient. The number of ASVs, Shannon and Chao1 indices were correlated with each environmental factor by Mantel test.

Based on a 97% sequence similarity threshold, 10,535 ASVs were obtained for each sample after normalization. The coverage index of the bacterial ASVs was greater than 0.999, indicating that the sequencing depth was reasonable. Compared with CK, drought decreased the Shannon index in the rhizosphere bacterial community of *N. tangutorum*, while salt stress increased the number of ASVs and the Chao1 index but not significantly (Supplementary Table 2). The rhizosphere soil bacterial community diversity of *N. tangutorum* was significantly correlated with soil SWC, TN, and SOM, and SWC and TN were positively correlated. The correlation between pH and EC was stronger under drought stress, whereas that between SWC and EC was stronger under salt stress. The ASVs and Shannon and Chao1 indices of the rhizosphere soil bacterial community of *N. tangutorum* were correlated with soil pH and EC under salt stress (*p* < 0.05; Figure 1). CCA analysis showed that Firmicutes and Bacteroidetes were correlated with soil SOM under drought stress; while Acidobacteriota, Chloroflexi, Proteobacteria, Actinobacteriota, and Gemmatimonadota were correlated with soil SWC, TS, and Na^+^. The analysis explained 27.95 and 14.11% of the variables, respectively (Supplementary Figure 1).

### Rhizosphere soil bacterial community composition and r-and k-strategists under drought and salt stress

**Figure 2:**
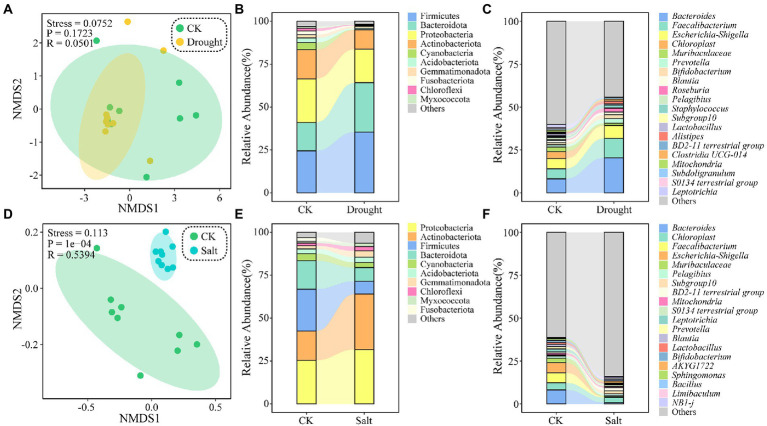
Non-metric multidimensional scaling (NMDS) ordination based on Bray Curtis similarities of bacterial communities under drought **(A)** and salt **(D)** stress. Each point represents a replicate, different colors indicate different sample plots. Relative abundance of major taxa (top 10) in the bacterial communities under drought **(B)** and salt **(E)** stress at the phylum level. And relative abundance of major taxa (top 20) in the bacterial communities under drought **(C)** and salt **(F)** stress at the genera level.

Non-metric multidimensional scale analysis revealed that the rhizosphere soil bacterial community was clustered under drought stress but dispersed under salt stress. The ASVs of *N. tangutorum* rhizosphere soil bacteria were classified into 50 phyla and 999 genera. The top 10 phyla in relative abundance under drought stress were Firmicutes, Bacteroidota, Proteobacteria, Actinobacteriota, Cyanobacteria, Acidobacteriota, Gemmatimonadota, Fusobacteriota, Chloroflexi, and Myxococcota. Compared with that of CK, the abundance of Proteobacteria (25.44–19.55%) and Actinobacteriota (17.02–11.19%) decreased, whereas that of Firmicutes (24.45–35.44%) and Bacteroidota (16.56–28.78%) increased under drought stress. When considering the relative abundance under salt stress, the top 10 phyla were Proteobacteria, Actinobacteriota, Firmicutes, Bacteroidota, Cyanobacteria, Acidobacteriota, Gemmatimonadota, Chloroflexi, Myxococcota, and Fusobacterium. Under salt stress, the abundance of Proteobacteria (25.44–31.70%) and Actinobacteriota (17.02–32.36%) increased, while Firmicutes (24.45–7.40%) and Bacteroidota decreased (16.56–8.01%) compared with that in CK. The relative abundance of Cyanobacteria decreased under drought and salt stress conditions. Thus, the relative abundance of *Bacteroides*, *Faecalibacterium,* and *Escherichia-Shigella* decreased under drought and salt stress (Figure 2).

**Figure 3:**
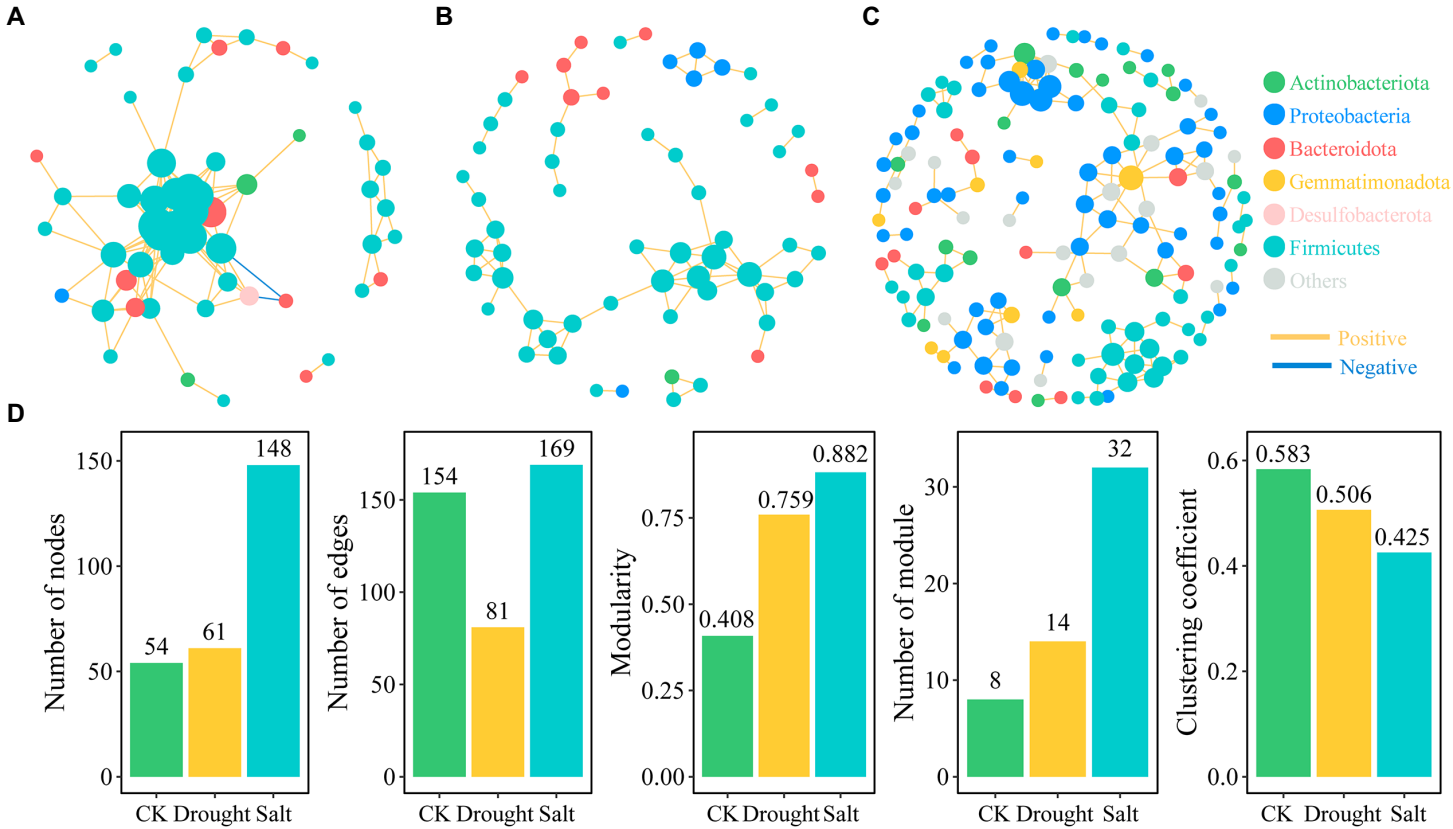
The relative abundance of bacteria for r-and k-strategists **(A,B)** and the correlation with rhizosphere soil properties of *N*. *tangutorum* under different stress **(C,D)**. Drought: **A** and **C**; Salt: **B** and **D**. “*” represents *p* < 0.05, “**” represents *p* < 0.01, and “***” represents *p* < 0.001.

There were two r-strategist bacteria (Gammaproteobacteria and Bacteroidota) and two k-strategists (Alphaproteobacteria and Acidobacteriota) in our study. The relative abundance of Gammaproteobacteria (15.65–16.03%) and Bacteroidota (16.56–28.78%) as r-strategists increased, whereas that of Alphaproteobacteria (9.78–3.52%) and Acidobacteriota (2.70–0.76%) as k-strategists decreased under drought stress compared with that of CK. The relative abundance of Gammaproteobacteria (15.65–10.84%) and Bacteroidota (16.56–8.01%) as r-strategists decreased, whereas Alphaproteobacteria (9.78–20.86%) and Acidobacteriota (2.70–3.06%) as k-strategists increased under salt stress (Figures 3A,B). Gammaproteobacteria were significantly negatively correlated with TC under drought stress; furthermore, Gammaproteobacteria, Acidobacteriota, and Bacteroidota were significantly negatively correlated with TC under salt stress. Gammaproteobacteria and Acidobacteriota were significantly negatively correlated with TN, and Acidobacteriota was significantly negatively correlated with SOM under salt stress (*p* < 0.05; Figure 3).

### Rhizosphere soil bacterial network under drought and salt stress

**Figure 4:**
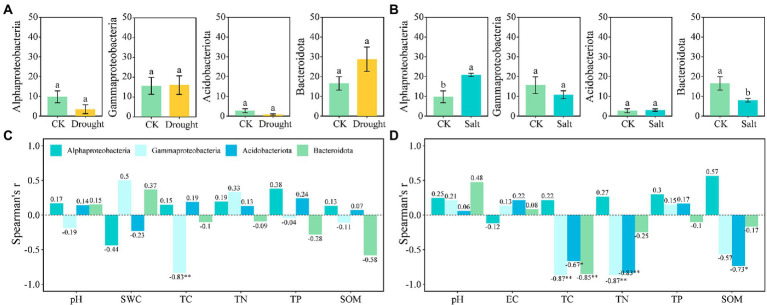
Bacterial networks based on correlation analysis of *Nitraria tangutorum* rhizosphere soil under different stress (**A**: CK; **B**: Drought; and **C**: Salt) and the correlation between network properties and soil properties **(D)**. A connection stands for a strong (Spearman’s|r|> 0.9) and significant (*p* < 0.01 after FDR correction). Colours of nodes indicate different major phyla while node size is proportional to node degree calculated from each ASV abundance correlation. The yellow lines indicate positive interactions whereas blue lines indicate negative interactions. **(D)** The network topological features of *N.tangutorum* under drought and salt stress.

Based on correlation analysis (*p* < 0.01), we constructed bacterial co-occurrence networks of *N. tangutorum* under CK, drought, and salt stress. The co-occurrence network showed that drought and salt stress decreased ASV aggregation in the rhizosphere soil bacterial community of *N. tangutorum*. Under drought stress, the connectivity and complexity of the rhizosphere soil bacterial community network of *N. tangutorum* and the number of edges (47.4%) decreased compared with those of CK. In contrast, salt stress increased the connectivity and complexity of the bacterial community network, and the number of nodes and edges increased by 174.07 and 9.74%, respectively, compared with those of CK (Figure 4). There were 54 nodes and 154 edges, of which 152 were positive and 2 were negative, for CK. Under drought and salt stress, the number of nodes were 61 and 148, number of edges were 81 and 169, respectively, and all were positive. The number of modules was highest (32) under salt stress, followed by those under drought stress (14) and CK (8). Topological features such as graph density, clustering coefficient, betweenness centralization, and degree centralization were the highest in CK, followed by drought stress, and the lowest in salt stress (Supplementary Table 3).

### Correlation among root exudates, r-and k-strategists bacteria, and network properties under drought and salt stress

**Figure 5:**
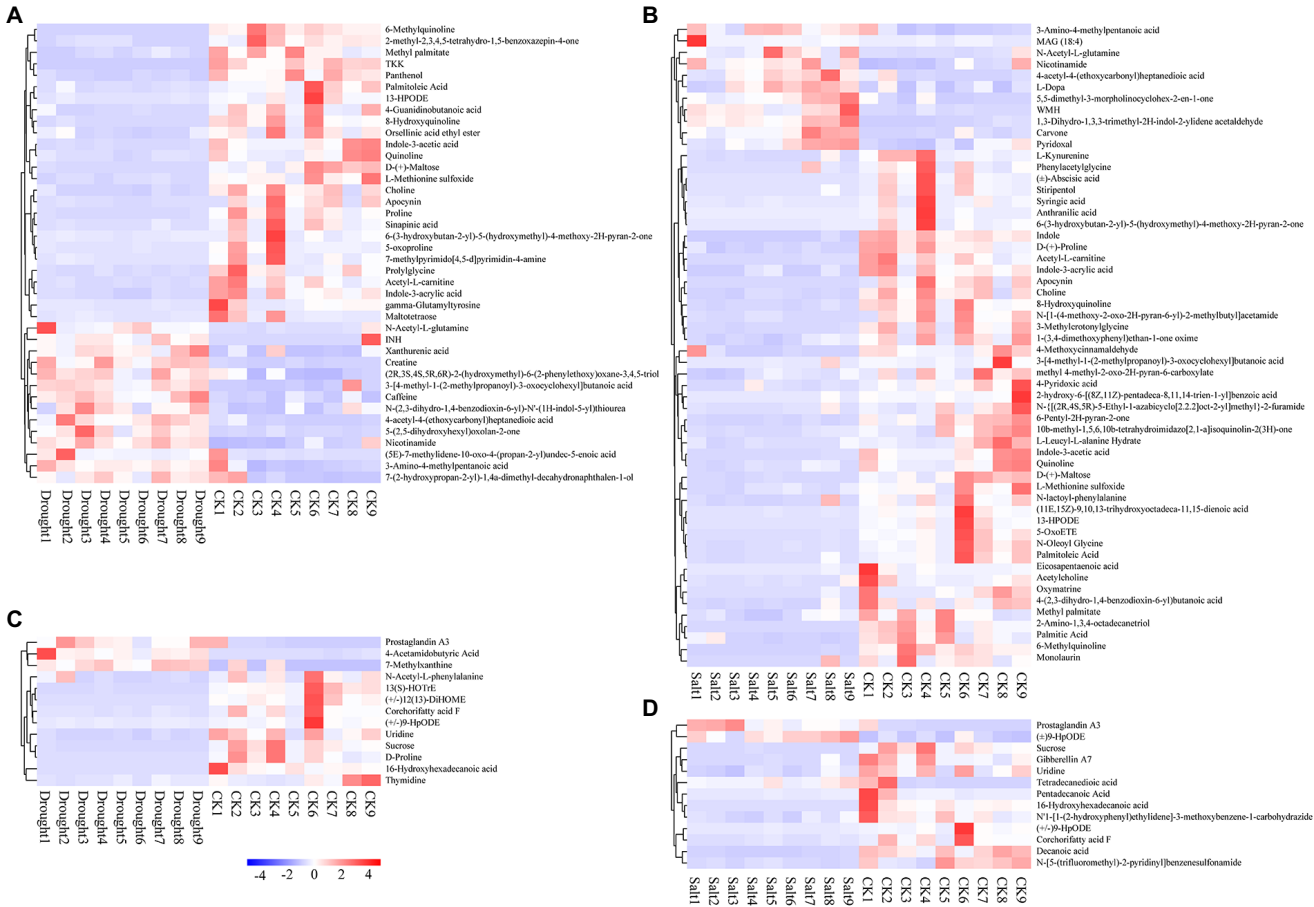
Heat map of clustering of differential metabolites of *Nitraria tangutorum* under drought (**A**: pos and **C**: neg) and salt (**B**: pos and **D**: neg) stress.

The characteristic peaks of the chromatograms of *N. tangutorum* root exudates under different stress conditions were markedly different. By preprocessing the raw data and comparing the database, 152 and 60 compounds in positive and negative ion modes, respectively, were identified. Root exudates contained various organic acids, amides, esters, sugars, olefins, phenols, growth factors, aromatics, ketones, amino acids and their derivatives, and heterocyclic compounds. Most of the exudates were secondary metabolites. Significant differences in metabolites of *N. tangutorum* root systems under drought and salt stress, with most metabolites correlated with environmental changes (Supplementary Figures 2, 3). Under drought stress, there were 40 differential metabolites in the positive ion mode, of which 14 were upregulated and 26 were downregulated. In addition, there were 13 differential metabolites in the negative ion mode, of which three were upregulated and 10 were downregulated. Under salt stress, there were 56 differential metabolites in the positive ion mode, of which 11 were upregulated and 45 were downregulated. However, in the negative ion mode, there were 13 differential metabolites, of which three were upregulated and 10 were downregulated (Figure 5).

**Figure 6:**
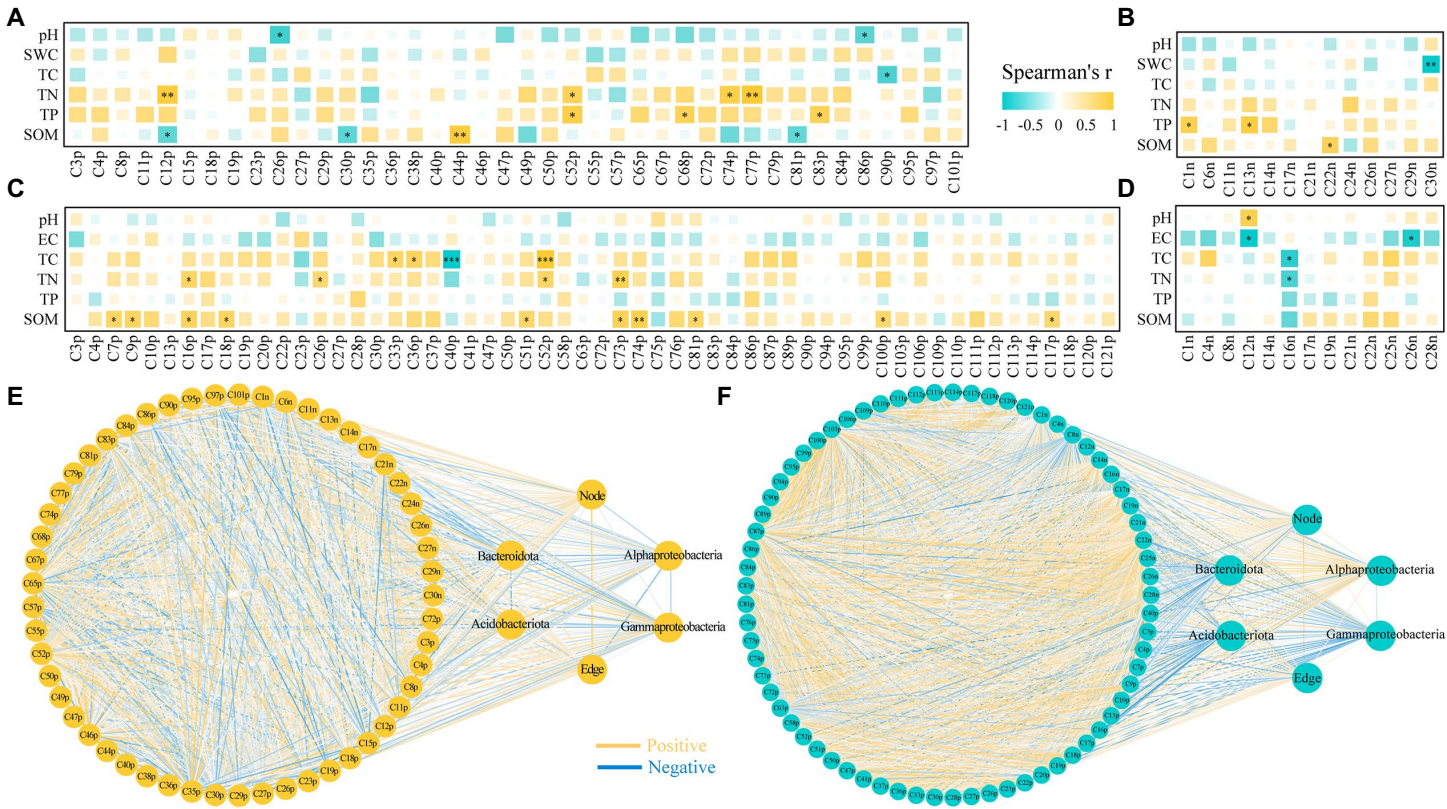
Correlation between root exudates and rhizosphere soil properties, bacteria for r-and k-strategists and network properties of *Nitraria tangutorum* under drought and salt stress. **(A)** Spearman correlation coefficients between differential metabolites of positive ion mode and rhizosphere soil physicochemical properties under drought stress. **(B)** Spearman correlation coefficients between differential metabolites of negative ion mode and rhizosphere soil physicochemical properties under drought stress. **(C)** Spearman correlation coefficients between differential metabolites of positive ion mode and rhizosphere soil physicochemical properties under salt stress. **(D)** Spearman correlation coefficients between differential metabolites of negative ion mode and rhizosphere soil physicochemical properties under salt stress. Correlation between differential metabolites of positive and negative ion mode, bacteria for r-and k-strategists and network properties of *N. tangutorum* under drought **(E)** and salt **(F)** stress. The significance of factors and the correlations between factors is marked at the figure, where “*” represents *p* < 0.05, “**” represents *p* < 0.01, and “***” represents *p* < 0.001; yellow lines indicate positive correlations whereas blue lines indicate negative correlations. Name of root exudates under drought stress: C50p: 6-Methylquinoline; C68p: TKK; C3p: Proline; C38p: Creatine; C67p: 8-Hydroxyquinoline; C86p: Panthenol; C19p: Indole-3-acetic acid; C95p: D-(+)-Maltose; C29p: 5-(2,5-dihydroxyhexyl)oxolan-2-one; C27p: Prolylglycine; C4p: Choline; C90p: 2-methyl-2,3,4,5-tetrahydro-1,5-benzoxazepin-4-one; C18p: Acetyl-L-carnitine; C15p: L-Methionine sulfoxide; C26p: Indole-3-acrylic acid; C35p: 4-Guanidinobutanoic acid; C79p: 4-acetyl-4-(ethoxycarbonyl) heptanedioic acid; C55p: Methyl palmitate; C40p: Palmitoleic Acid; C36p: 13-HPODE; C101p: Sinapinic acid; C97p: 5-oxoproline; C77p: 7-methylpyrimido [4,5-d]pyrimidin-4-amine; C47p: Quinoline; C11p: Nicotinamide; C52p: 3-[4-methyl-1-(2-methylpropanoyl)-3-oxocyclohexyl] butanoic acid; C12p: Caffeine; C57p: N-Acetyl-L-glutamine; C65p: gamma-Glutamyltyrosine; C8p: Apocynin; C84p: Orsellinic acid ethyl ester; C83p: INH; C30p: Xanthurenic acid; C72p: (2R,3S,4S,5R,6R)-2-(hydroxymethyl)-6-(2-phenylethoxy)oxane-3,4,5-triol; C46p: (5E)-7-methylidene-10-oxo-4-(propan-2-yl)undec-5-enoic acid; C44p: 3-Amino-4-methylpentanoic acid; C81p: Maltotetraose; C23p: 7-(2-hydroxypropan-2-yl)-1,4a-dimethyl-decahydronaphthalen-1-ol; C49p: N-(2,3-dihydro-1,4-benzodioxin-6-yl)-N′-(1H-indol-5-yl)thiourea; C74p: 6-(3-hydroxybutan-2-yl)-5-(hydroxymethyl)-4-methoxy-2H-pyran-2-one; C1n: Sucrose; C27n: 13(S)-HOTrE; C22n: 16-Hydroxyhexadecanoic acid; C21n: (+/−)12 (13)-DiHOME; C13n: 4-Acetamidobutyric Acid; C14n: D-Proline; C26n: Uridine; C29n: Corchorifatty acid F; C24n: Prostaglandin A3; C17n: (+/−)9-HpODE; C11n: 7-Methylxanthine; C6n: N-Acetyl-L-phenylalanine; and C30n: Thymidine. Name of root exudates under salt stress: C99p: Apocynin; C113p: (±)-Abscisic acid; C40p: 2-Amino-1,3,4-octadecanetriol; C106p: (11E,15Z)-9,10,13-trihydroxyoctadeca-11,15-dienoic acid; C13p: 8-Hydroxyquinoline; C19p: Indole-3-acetic acid; C36p: 5-OxoETE; C90p: 3-Methylcrotonylglycine; C18p: Acetyl-L-carnitine; C26p: D-(+)-Maltose; C120p: Indole; C27p: 6-Methylquinoline; C47p: WMH; C37p: 13-HPODE; C86p: N-Oleoyl Glycine; C3p: Choline; C83p: Oxymatrine; C50p: 6-Pentyl-2H-pyran-2-one; C109p: N-[1-(4-methoxy-2-oxo-2H-pyran-6-yl)-2-methylbutyl]acetamide; C84p: L-Kynurenine; C75p: Eicosapentaenoic acid; C33p: Palmitoleic Acid; C4p: D-(+)-Proline; C114p: 2-hydroxy-6-[(8Z,11Z)-pentadeca-8,11,14-trien-1-yl] benzoic acid; C94p: 4-(2,3-dihydro-1,4-benzodioxin-6-yl) butanoic acid; C20p: Syringic acid; C52p: Quinoline; C23p: Palmitic Acid; C9p: Carvone; C87p: Stiripentol; C89p: 4-Pyridoxic acid; C72p: Monolaurin; C22p: 1,3-Dihydro-1,3,3-trimethyl-2H-indol-2-ylidene acetaldehyde; C110p: 1-(3,4-dimethoxyphenyl)ethan-1-one oxime; C74p: 5,5-dimethyl-3-morpholinocyclohex-2-en-1-one; C17p: L-Methionine sulfoxide; C100p: 4-acetyl-4-(ethoxycarbonyl) heptanedioic acid; C28p: Acetylcholine; C103p: N-lactoyl-phenylalanine; C30p: Indole-3-acrylic acid; C118p: 3-Amino-4-methylpentanoic acid; C51p: Anthranilic acid; C63p: 4-Methoxycinnamaldehyde; C81p: 3-[4-methyl-1-(2-methylpropanoyl)-3-oxocyclohexyl] butanoic acid; C58p: L-Leucyl-L-alanine Hydrate; C117p: Phenylacetylglycine; C76p: N-Acetyl-L-glutamine; C16p: methyl 4-methyl-2-oxo-2H-pyran-6-carboxylate; C112p: 10b-methyl-1,5,6,10b-tetrahydroimidazo [2,1-a]isoquinolin-2(3H)-one; C111p: N-{[(2R,4S,5R)-5-Ethyl-1-azabicyclo [2.2.2] oct-2-yl]methyl}-2-furamide; C10p: Nicotinamide; C95p: 6-(3-hydroxybutan-2-yl)-5-(hydroxymethyl)-4-methoxy-2H-pyran-2-one; C73p: L-Dopa; C41p: Methyl palmitate; C7p: Pyridoxal; C121p: MAG (18: 4); C1n: Sucrose; C4n: Uridine; C8n: N-[5-(trifluoromethyl)-2-pyridinyl]benzenesulfonamide; C12n: (+/−)9-HpODE; C14n: Decanoic acid; C16n: Prostaglandin A3; C17n: N′1-[1-(2-hydroxyphenyl)ethylidene]-3-methoxybenzene-1-carbohydrazide; C19n: 16-Hydroxyhexadecanoic acid; C21n: Tetradecanedioic acid; C22n: (±)9-HpODE; C25n: Pentadecanoic Acid; C26n: Corchorifatty acid F; and C28n: Gibberellin A7.

Under drought stress, indole-3-acrylic acid and panthenol were negatively correlated with pH; 2-methyl-2,3,4,5-tetrahydro-1,5-benzoxazepin-4-one and thymidine were negatively correlated with SWC; caffeine, 6-(3-hydroxybutan-2-yl)-5-(hydroxymethyl)-4-methoxy-2H-pyran-2-one, and 7-methylpyrimido [4,5-d]pyrimidin-4-amine were positively correlated with TN; 3-[4-methyl-1-(2-methylpropanoyl)-3-oxocyclohexyl] butanoic acid, TKK, INH, sucrose, and 4-acetamidobutyric acid were positively correlated with TP; caffeine, xanthurenic acid, and maltotetraose were negatively correlated with SOM; and 3-amino-4-methylpentanoic acid and 16-hydroxyhexadecanoic acid were positively correlated with SOM. Under salt stress, (+/−)9-HpODE was negatively correlated with pH; (+/−)9-HpODE and corchorifatty acid F were negatively correlated with EC; palmitoleic acid, 5-OxoETE, and quinoline were positively correlated with TC; 2-amino-1,3,4-octadecanetriol and prostaglandin A3 were negatively correlated with TC; methyl 4-methyl-2-oxo-2H-pyran-6-carboxylate, D-(+)-maltose, quinoline, and L-dopa were positively correlated with TN; prostaglandin A3 was negatively correlated with TN; pyridoxal, carvone, methyl 4-methyl-2-oxo-2H-pyran-6-carboxylate, acetyl-L-carnitine, anthranilic acid, L-dopa, 5,5-dimethyl-3-morpholinocyclohex-2-en-1-one, 3-[4-methyl-1-(2-methylpropanoyl)-3-oxocyclohexyl] butanoic acid, 4-acetyl-4-(ethoxycarbonyl) heptanedioic acid, and phenylacetylglycine were positively correlated with SOM (Figure 6).

## Discussion

### Drought and salt stress caused significant differences in rhizosphere soil physicochemical properties and root exudates of *Nitraria tangutorum*

Soil water and salt are important factors affecting soil physicochemical properties and plant growth (Flowers et al., 2010). Our study focused on the rhizosphere soil physicochemical characteristics and *N. tangutorum* bacterial community assembly strategies under short-term drought and salt stress conditions in a desert steppe. The results showed that under short-term drought stress, rhizosphere soil pH increased significantly with a decrease in SWC, while EC and pH increased significantly under salt stress. Li et al. (2018) and Pan et al. (2021) concluded that gradient changes in SWC and pH affect soil physicochemical properties, particularly the accumulation of TC content. Although the effect of short-term stress on TOC content was not significant, our study found that drought and salt stress reduced the TOC content of the rhizosphere soil of *N. tangutorum*. Dong et al. (2019) also pointed out that soil TOC is mainly controlled by soil salt concentration, further supporting our conclusions.

Plant roots respond to abiotic stress by secreting exudates, including amino acids, carbohydrate peptides, and phenolic compounds (Mercado-Blanco and Bakker, 2007). Under drought stress, 4-acetyl-4-(ethoxycarbonyl) heptanedioic, N-acetyl-L-glutamine, xanthurenic, and 4-acetamidobutyric acids accumulated in the root exudates of *N. tangutorum*. Elevated phenolic acid content may be a strategy to cope with prolonged plant growth and stress (Henry et al., 2007). As an important metabolite of plant roots, phenolic acids play an important role in regulating their biological functions (Venugopal et al., 2009). We also found that growth factors such as creatine and nicotinamide accumulate during drought stress. These substances are secondary plant metabolites that play a crucial role in plant defense and adaptation to environmental stresses (Fujii et al., 2012). In our study, the D-proline and sucrose contents decreased significantly after 30 days of drought, differing from previous studies (Gong et al., 2005). Plants improve root osmoregulation through changes in sucrose and polyol metabolism under stress (Ruan, 2014). The decrease may be caused by the longer treatment time in our study than in previous studies. However, after 30 days of salt stress, D-proline content in *N. tangutorum* root exudates increased substantially, further proving that the difference in the amount and type of plant root exudates was due to different response strategies under different stressful environments (Per et al., 2017).

In our study, rhizosphere soil pH and EC were mostly negatively correlated with *N. tangutorum* root exudates under drought and salt stress, indicating that the unbalanced absorption and utilization of soil cations and anions by plants under stress resulted in the accumulation of organic and phenolic acids in the root exudates, which in turn had a strong correlation with rhizosphere soil pH (Gregory, 2007). In addition, the root exudates of plants positively correlated with soil TC, TN, TP, and SOM, indicating that root exudates affected plant nutrient uptake, especially in stressful environments. Root exudates have been shown to indirectly affect soil accumulation by secreting carbon-containing organic compounds and low molecular organic acids, which affect the microbial population and enzyme activity related to soil nutrients (Narula et al., 2008). In our study, 3-[4-methyl-1-(2-methylpropanoyl)-3-oxocyclohexyl] butanoic acid, quinoline, L-dopa, and 7-methylpyrimido [4,5-d] pyrimidin-4-amine were strongly correlated with rhizosphere soil TC, TN, and TP content, confirming that root exudates are an important vehicle for material exchange between plant roots and the soil (Santhanam et al., 2015).

### Drought and salt stress changed the rhizosphere soil bacterial community structure of *Nitraria tangutorum*

Changes in soil physicochemical properties significantly affect soil bacterial community structure and diversity (Orozco-Aceves et al., 2017; Huang et al., 2019). However, in the present study, there were no significant differences in the abundance and diversity of *N. tangutorum* rhizosphere soil bacteria under drought and salt stress compared with those in CK, indicating that the rhizosphere bacterial community structure was stable, consistent with the results of Liu et al. (2021). NMDS analysis showed that the bacterial community structure of *N. tangutorum* rhizosphere soil was similar under drought and CK (Figure 2), which might be caused by a single species and limited diffusion of the bacterial community. Salt stress changes the composition of the soil bacterial community. Salt induced changes in the *N. tangutorum* rhizosphere bacterial community more than drought, reflecting the response characteristics of plant rhizosphere microorganisms to salt stress (Sarkar et al., 2018; Tiepo et al., 2018). In the current study, Firmicutes, Bacteroidota, Proteobacteria, and Actinobacteriota were the dominant phyla in the *N. tangutorum* rhizosphere. Previous studies have indicated that these bacteria are enriched in the rhizosphere of plants (Filion et al., 2004; Sanguin et al., 2006; Lee et al., 2008; Xu et al., 2009; Gottel et al., 2011;). Under drought stress, the relative abundance of Firmicutes increases, probably because the genera of this phylum are single-skinned bacteria with a thick cell wall that has better resistance to water stress (Lennon et al., 2012; Naylor et al., 2017; Cregger et al., 2018; Xu et al., 2018). However, the relative abundance of Proteobacteria and Actinobacteriota increased under salt stress. The sporogenesis ability of Actinobacteriota has been suggested to enable them to remain stable and quiescent in stressful environments, a strategy that may enable them to survive under adverse conditions (Vanessa et al., 2013; Hacquard et al., 2015; Naylor et al., 2017). Furthermore, a lack of nutrients in the plant rhizosphere soil would increase the abundance of Firmicutes (Singh et al., 2007). In our study, the plants were grown in sandy soils with poor fertility, which was exacerbated by drought. In the present study, drought and salt treatments may have caused drastic changes in soil moisture and pH, affecting the composition of microbial communities. The coexistence of microbial species usually depends on metabolic trade-offs, with each species having an adaptive advantage under specific biotic and abiotic conditions (Xu et al., 2018). *Bacteroides* and *Faecalibacterium* in *N. tangutorum* rhizosphere soil, belonging to phylum Bacteroidota, showed similar changes under drought and salt stress. Studies have shown that the relative abundance of bacteria responding to stress consistently changes for some phyla, classes, orders, families, and genera, which may reflect the common functions and life strategies of specific bacterial lineages (Philippot et al., 2010; Amend et al., 2016). Plants or host species growing in the same environment can attract and aggregate different microbial communities in the root zone and rhizosphere (Aleklett et al., 2015; Samad et al., 2017). Changes in plant rhizosphere soil bacteria observed under drought conditions are usually associated only with changes in drought-sensitive bacterial species but not with changes in the overall plant microflora (Naylor and Coleman-Derr, 2018). Therefore, we hypothesized that the stable rhizosphere soil bacterial diversity of *N. tangutorum* under drought stress is due to the increased abundance of specific bacteria in the rhizosphere soil. Bacterial community network analysis showed that the degree of isolation in the operational taxonomic unit module of the rhizosphere soil core bacterial community of *N. tangutorum* under drought stress was significantly higher than that in CK (Figure 4).

### Salt stress changed the interaction of rhizosphere soil bacteria and the r-and k-strategists of *Nitraria tangutorum*

Under drought and salt stress, plants can influence the species and quantity of bacterial communities in the rhizosphere through changes in root exudates (Mayak et al., 2004). The rhizosphere soil bacterial community structure of *N. tangutorum* under drought and salt stress was significantly different. In stressful environments, plant rhizosphere actively release large amounts of bioactive substances, sugars, and organic acids to attract functional bacteria for colonization, thereby changing the structure of the rhizosphere microbial community to adapt to stress (Fierer, 2017; Keswani et al., 2019). In addition, root exudate composition plays a selective role in establishing rhizosphere microbial communities (Wieland et al., 2001; Kowalchuk et al., 2002). Zwetsloot et al. (2020) found that ubiquitous root phenols could alter soil microbial communities. Changes in soil chemical properties and structure caused by rhizosphere metabolic activities allow growth of specific rhizosphere microbial communities (Haichar et al., 2008; Chaparro et al., 2014; Lareen et al., 2016). Our results further confirmed previous conclusions and also found that the root metabolites of plants under different stresses shaped microbial communities with different ecological functions.

For the ecological functions, microbial taxa are often divided into r-and k-strategists based on their growth, reproduction, competition, and adaptation strategies (Fierer et al., 2007; Li et al., 2021). The observed differences in rhizosphere bacterial populations of r-and k-strategists in *N. tangutorum* under salt stress shed light on the potential microbial mechanism of plant root exudates regulating the interactions among rhizosphere bacteria under stressful environments. Numerous studies have shown that r-strategist bacteria grow and reproduce rapidly in nutrient-rich environments, whereas k-strategist bacteria grow slowly under oligotrophic conditions (Fierer et al., 2007). In the present study, Bacteroidota and Alphaproteobacteria showed r-and k-strategist characteristics, respectively. Under salt stress, plants select suitable bacteria for colonization using different root exudates and then change the bacterial community structure. It has been confirmed that soil bacterial communities are usually limited by the availability of carbon substrates (Raich and Tufekciogul, 2000). In the current study, Bacteroidota and Alphaproteobacteria showed stronger correlations with TC and SOM under salt stress. Therefore, it can be inferred that salt stress reduced soil nutrient availability and inhibited the growth of r-strategist bacteria (Sicardi et al., 2004), whereas the accumulation of unstable carbon sources in low-molecular-weight compounds in *N. tangutorum* root exudates increased the k-strategist bacterial community (Kielak et al., 2009; Zhang et al., 2018).

The high connectivity of soil bacterial network relationships is considered a rapid response to environmental changes (Zhang et al., 2018). Many studies have shown that the stronger the plant-microbe interactions in the soil, the stronger the network relationships (Zhang et al., 2018). We found that rhizosphere soil bacteria had a closer network relationship under CK, whereas drought stress reduced the network connectivity and complexity of *N. tangutorum* rhizosphere soil bacteria. Interactions among the rhizosphere soil bacteria of *N. tangutorum* also decreased under salt stress. Furthermore, Shi et al. (2016) pointed out that the connectivity and complexity of soil microbial networks are important indicators of soil physicochemical properties that determine plant-bacteria interactions (Shi et al., 2016; Li et al., 2018). In our study, the number of nodes and edges in the bacterial network changed with soil moisture and salinity, indicating that these changes were influenced by the soil physicochemical properties. Guan et al. (2021) reported that simple network relationships could negatively affect geochemical functions, especially in salinized soils with unstable bacterial communities. Our study confirms the above conclusion that soil bacterial interactions gradually decrease with increasing soil EC and pH. However, changes in soil physicochemical properties caused by rhizosphere metabolism can shape specific rhizosphere microbial communities (Haichar et al., 2008; Chaparro et al., 2014; Lareen et al., 2016). Our results also confirmed that a change in root exudates caused by soil salinity affects the interaction among rhizosphere soil bacteria.

## Conclusion

In conclusion, the rhizosphere soil bacterial community of *N. tangutorum* was highly responsive to environmental changes (drought and salt stress). Drought and salt stress decreased the nutrient content of the rhizosphere soil of *N. tangutorum*, while the differential metabolites such as organic acids, growth hormones, and sugars were the active strategies for the root system in response to stress. Meanwhile, the correlation between bacterial community diversity, richness and soil physicochemical properties was enhanced, which drove the bacterial community towards k-strategists. The network analysis also highlighted the effect of environmental changes on rhizosphere bacterial interactions, while differential root metabolites were important factors influencing the r/k categories of bacterial communities. Our analysis further suggests that root exudates play a prominent role as life carriers between plants and the soil environment in responding to environmental changes and assembling the structure of the rhizosphere microbial communities. This study reveals the mechanism of plant–soil-microbe interactions under the action of root exudates, and provides new ideas for studying the response of bacterial communities to stressful environments.

## Data availability statement

The datasets presented in this study can be found in online repositories. The names of the repository/repositories and accession number(s) can be found at: https://www.ncbi.nlm.nih.gov/, PRJNA855333.

## Author contributions

PK and NS conceived and designed the study. YP, MT, JH, and YZ did running the experiments and data management. JH and YP performed the data mining, statistical analysis, interpretation, and figure and table preparation of the 16S rRNA amplicon sequencing results. YP, PK, JZ, and XL did the manuscript writing and revising. All authors contributed to the article and approved the submitted version.

## Funding

This work was supported financially by the National Natural Science Foundation of China (41621001), Key Research and Development Project of Ningxia (2019BEB04018), Ningxia Natural Science Foundation (2022AAC03227), and Innovation Team for Genetic Improvement of Economic Forests Foundation in Ningxia (2022QCXTD04).

## Conflict of interest

The authors declare that the research was conducted in the absence of any commercial or financial relationships that could be construed as a potential conflict of interest.

## Publisher’s note

All claims expressed in this article are solely those of the authors and do not necessarily represent those of their affiliated organizations, or those of the publisher, the editors and the reviewers. Any product that may be evaluated in this article, or claim that may be made by its manufacturer, is not guaranteed or endorsed by the publisher.

## Footnotes

^1^ https://ccb.jhu.edu/software/FLASH/index.shtml

^2^ https://github.com/Hy4m/linkET

## Abbreviations

CK: Control samples
SWC: Soil water content
EC: Electrical conductivity
TC: Soil total carbon
TN: Total nitrogen
TP: Total phosphorus
TOC: Soil total organic carbon
NMDS: Non-metric multidimensional scale
QC: Quality control
LC–MS: Liquid chromatography-mass spectrometry
SOM: Soil organic matter
