# Supplementary material for: Root exudates and rhizosphere soil bacterial relationships of Nitraria tangutorum are linked to k-strategists bacterial community under salt stress
Source: Front Plant Sci. 2022 Aug 31;13:997292. doi: 10.3389/fpls.2022.997292 (PMC9471988; doi:10.3389/fpls.2022.997292)
Supplement: Supplementary file 1 [file Data_Sheet_1.docx]

**Supplementary Figure 1** Canonical correlation analysis of differential metabolites and rhizosphere soil physicochemical properties of *N*. *tangutorum* under drought and salt stress.

**Supplementary Figure 2** Canonical correlation analysis of differential metabolites and rhizosphere soil physicochemical properties of *N*. *tangutorum* under drought (A) and salt (B) stress.

**Supplementary Figure 3** Positive and negative mode of differential metabolites from *N*. *tangutorum* root exudates under drought (A and B) and salt (C and D) stress. Red points represent up-regulated and green points represent down-regulated.
