# Supplementary material for: Root exudates and rhizosphere soil bacterial relationships of Nitraria tangutorum are linked to k-strategists bacterial community under salt stress
Source: Front Plant Sci. 2022 Aug 31;13:997292. doi: 10.3389/fpls.2022.997292 (PMC9471988; doi:10.3389/fpls.2022.997292)
Supplement: Supplementary file 2 [file Data_Sheet_2.docx]

**Supplementary Table 1** Physicochemical properties of *N.tangutorum* rhizosphere soil under drought and salt stress

|  | CK | Drought stress | Salt stress |
| --- | --- | --- | --- |
| SWC (%) | 19.78±1.58a | 7.68±0.52b | 19.95±1.43a |
| pH | 7.62±0.08c | 8.33±0.08b | 9.19±0.09a |
| EC (us/cm) | 43.11±2.77b | 59.33±3.43b | 479.11±34.36a |
| TC (g/kg) | 8.48±0.56a | 5.55±0.44b | 5.94±0.32b |
| TN (g/kg) | 0.28±0.03a | 0.21±0.02a | 0.26±0.05a |
| TP (g/kg) | 0.35±0.03a | 0.28±0.02b | 0.25±0.02b |
| SOM (g/kg) | 6.66±0.87a | 4.83±0.31a | 4.57±0.97a |
| NH+ 4-N (mg/kg) | 2.38±0.20a | 1.46±0.17b | 2.13±0.11a |
| NO- 3-N (mg/kg) | 7.40±1.03b | 10.93±1.20b | 24.75±1.44a |

**Supplementary Table 2** Alpha diversity of *N.tangutorum* under drought and salt stress

|  | CK | Drought stress | Salt stress |
| --- | --- | --- | --- |
| ASVs | 810.78±222.59ab | 549.67±195.44b | 1120.89±70.94a |
| Shannon | 7.03±0.59a | 5.97±0.59a | 7.3±0.35a |
| Simpson | 0.96±0.02a | 0.93±0.03a | 0.95±0.01a |
| Chao1 | 813.73±222.77ab | 552.4±195.82b | 1123.54±71.33a |

**Supplementary Table 3** Network topological features of *N.tangutorum* under drought and salt stress

|  | CK | Drought | Salt |
| --- | --- | --- | --- |
| Nodes | 54 | 61 | 148 |
| Total edges | 154 | 81 | 169 |
| Positive edges (%) | 152(98.7) | 81(100) | 169(100) |
| Negative edges (%) | 2(1.3) | 0(0) | 0(0) |
| Number of modules | 8 | 14 | 32 |
| Average Path length | 2.96 | 4.21 | 3.32 |
| Modularity | 0.41 | 0.76 | 0.88 |
| Clustering Coefficient | 0.58 | 0.51 | 0.43 |
| Density | 0.079 | 0.017 | 0.005 |
| Diameter | 8.42 | 10.13 | 10.23 |
| Betweenness centralization | 0.114 | 0.05 | 0.004 |
| Degree centralization | 0.18 | 0.07 | 0.03 |
